# Spiritual well-being, symptoms and performance of patients under palliative care

**DOI:** 10.1590/0034-7167-2022-0007

**Published:** 2023-04-07

**Authors:** Bárbara Vitória Mendes, Suzana Cristina Teixeira Donato, Thaina Lúcio da Silva, Ramon Moraes Penha, Paula Jaman-Mewes, Marina de Góes Salvetti

**Affiliations:** I Hospital Sírio Libanês. São Paulo, São Paulo, Brazil; II Hospital Nove de Julho. São Paulo, São Paulo, Brazil; III Hospital A.C. Camargo Cancer Center. São Paulo, São Paulo, Brazil; IV Universidade Federal do Mato Grosso do Sul. Campo Grande, Mato Grosso do Sul,, Brazil; V Universidad de los Andes. Santiago, Chile; VI Universidade de São Paulo. São Paulo, São Paulo, Brazil

**Keywords:** Palliative Care, Spirituality, Terminally Ill, Signs and Symptoms, Cross-Sectional Studies., Cuidados Paliativos, Espiritualidad, Enfermo Terminal, Signos y Síntomas, Estudios Transversales., Cuidados Paliativos, Espiritualidade, Doente Terminal, Sinais e Sintomas, Estudos Transversais.

## Abstract

**Objectives:**

to assess the relationship between spiritual well-being, symptoms and performance of patients under palliative care.

**Methods:**

this is a descriptive correlational study, conducted with 135 patients seen in palliative care outpatient clinics. Karnofsky Performance Status Scale, Edmonton Symptom Assessment Scale, Spirituality Scale and Hospital Anxiety and Depression Scale were used. Data were submitted to descriptive statistical analysis and Spearman’s correlation.

**Results:**

among participants, 68.2% were cancer patients. The most prevalent symptoms were changes in well-being (65.2%), anxiety (63.7%), sadness (63%) and fatigue (63%). Sadness, dyspnea, sleepiness, anxiety and depression presented weak to moderate correlation with spiritual well-being. Symptom overload showed weak negative correlation with performance.

**Conclusions:**

symptom intensification was correlated with worsening in spiritual well-being perception. The reduction in performance was related to increased number of symptoms, especially depression and anxiety.

## RESUMO

**Objetivos:**

avaliar a relação entre bem-estar espiritual, sintomas e funcionalidade de pacientes em cuidados paliativos.

**Métodos:**

estudo descritivo correlacional, realizado com 135 pacientes atendidos em ambulatórios de cuidados paliativos. A Escala Funcional de Karnofsky, a Escala de Avaliação de Sintomas de Edmonton, a Escala de Espiritualidade e a Escala Hospitalar de Ansiedade e Depressão foram utilizadas. Os dados foram submetidos à análise estatística descritiva e correlação de Spearman.

**Resultados:**

entre os participantes, 68,2% eram pacientes oncológicos. Os sintomas mais prevalentes foram alterações do bem-estar (65,2%), ansiedade (63,7%), tristeza (63%) e fadiga (63%). Tristeza, dispneia, sonolência, ansiedade e depressão apresentaram correlação fraca a moderada com bem-estar espiritual. A sobrecarga de sintomas mostrou correlação negativa fraca com funcionalidade.

**Conclusões:**

a intensificação dos sintomas esteve correlacionada à piora na percepção de bem-estar espiritual. A redução da funcionalidade esteve relacionada ao aumento da quantidade de sintomas, em especial depressão e ansiedade.

## RESUMEN

**Objetivos:**

evaluar la relación entre bienestar espiritual, síntomas y funcionalidad de pacientes en cuidados paliativos.

**Métodos:**

estudio descriptivo correlacional, realizado con 135 pacientes atendidos en consultas externas de cuidados paliativos. Se utilizaron la Escala Funcional de Karnofsky, la Escala de Evaluación de Síntomas de Edmonton, la Escala de Espiritualidad y la Escala de Ansiedad y Depresión Hospitalaria. Los datos fueron sometidos a análisis estadístico descriptivo y correlación de Spearman.

**Resultados:**

entre los participantes, 68,2% eran pacientes con cáncer. Los síntomas más prevalentes fueron cambios en el bienestar (65,2%), ansiedad (63,7%), tristeza (63%) y fatiga (63%). Tristeza, disnea, somnolencia, ansiedad y depresión mostraron una correlación débil a moderada con el bienestar espiritual. La carga de síntomas mostró una débil correlación negativa con la funcionalidad.

**Conclusiones:**

la intensificación de los síntomas se correlacionó con el empeoramiento de la percepción de bienestar espiritual. La reducción de la funcionalidad se relacionó con un aumento del número de síntomas, especialmente depresión y ansiedad.

## INTRODUCTION

The palliative care approach emphasizes the assessment and treatment of physical, psychosocial and spiritual symptoms, with a focus on alleviating suffering related to health problems. The World Health Organization (WHO) expanded the definition of palliative care after the proposed definition of the International Association for Hospice and Palliative Care (IHAPC), published in 2020, including the caregiver and emphasizing the importance of countries institute public policies for palliative care^(1)^. However, in the final phase of a serious disease, actions are directed primarily to physical signs and symptoms, with psychosocial and spiritual dimensions often underestimated.

The improvement and development of tools and strategies to improve palliative care practice in this field is emerging. To this end, it is important to know the relationship between symptoms and the spiritual well-being of these patients^(2)^.

Spirituality can be defined as the inner search for answers to the meaning and meaning of life, which may involve religious practices or not^(3)^. Puchalski^(4)^ defines spirituality in an international consensus as:

a dynamic and intrinsic aspect of humanity through which persons seek ultimate meaning, purpose, and transcendence, and experience relationship to self, family, others, community, society, nature, and the significant or sacred. Spirituality is expressed through beliefs, values, traditions, and practices.

Spiritual beliefs influence how people face diseases and the multidisciplinary team seems to play an important role in the investigation of spirituality, providing support to patients under palliative care and offering ways to integrate health care with spirituality^(5)^.

In palliative care, spirituality should be valued, as it has been identified as an important resource for coping with and optimizing the dying process, especially in questions related to the meaning of life^(2)^. In cancer patients, spirituality is an effective tool in reducing levels of anxiety and depression, leading to optimization of quality of life^(6)^.

Spiritual well-being considers a person’s subjective perception of their way of seeing and understanding life and the world. It translates personal openness to the integration of spirituality with other dimensions of life, promoting greater support and strengthening, generating improvement in coping skills^(7)^. Spiritual well-being involves a personal connection with something higher, which, when integrated with other dimensions of life, leads to strengthening and improving quality of life^(8)^.

When assessing interactions between spiritual well-being, uncomfortable symptoms, and quality of life in lung cancer patients undergoing treatment (n=132) in Korea, researchers identified causal relationships between spiritual well-being, loss of appetite, dyspnea, pain, and fatigue^(7)^. In Brazil, after analyzing the correlation between spiritual well-being and quality of life of elderly people undergoing hemodialysis treatment (n=169), a study identified that the construct of spiritual well-being was associated with quality of life^(8)^.

The search for spiritual well-being can happen in different ways, either through the practice of a religion, through the spirituality lived internally, through the practice of meditation, among other ways. There are instruments that propose to assess spiritual well-being, such as Functional Assessment of Chronic Illness Therapy-Spiritual Well-Being, a 12-item instrument, validated in Brazil, which has been used in several contexts and in a wide variety of cultures^(9)^.

Studies indicate that spiritual well-being is related to better quality of life and lower incidence of physical and emotional symptoms. A recent study in Finland showed a correlation between spiritual well-being and quality of life in patients with and without cancer eligible for palliative care^(10)^. Another cross-sectional study in Portugal, with 95 patients receiving palliative care, reported that spiritual well-being was significantly correlated with higher levels of physical, emotional and functional well-being, in addition to better quality of life^(11)^. However, when tracking the national literature on the subject, the number of studies still does not allow establishing an overview of trends, methodological models of investigation and standardization of instruments, among other variables. Thus, the present study envisages corroborating the national production in this direction as well.

Health teams need to reflect on how to respond to patients’ spiritual needs and overcome barriers to providing spiritual care, such as limited resources, lack of appropriate vocabulary, lack of time and weaknesses in training, as well as cultural or institutional factors^(2)^. Nursing professionals play an important role in managing the symptoms of patients under palliative care. Knowing the relationship between the presence of symptoms, spiritual well-being and performance can help to improve quality of care.

## OBJECTIVES

To assess the relationship between spiritual well-being, symptoms and performance of patients under palliative care.

## METHODS

### Ethical aspects

This study is part of a larger project entitled “*Validação psicométrica da versão brasileira do* Patient Dignity Inventory (PDI -Br)”^(12)^, submitted and approved by the Ethics Committee of the *Universidade de São Paulo* Nursing School. Upon agreeing to participate, patients signed an Informed Consent Form in two copies and answered the research instruments through an interview in a quiet and private environment.

### Study design, period, and location

This is a descriptive and correlational study, carried out at palliative care outpatient clinics of the *Instituto do Câncer do Estado de São Paulo* and the *Hospital das Clínicas* of the *Universidade de São Paulo* Medical School, from April to June 2018. This study followed the STROBE recommendation for cross-sectional studies^(13)^.

### Population, sample; inclusion and exclusion criteria

The population consisted of patients under palliative care in the outpatient context. The non-probabilistic sample included 135 patients undergoing palliative care, monitored at the above-mentioned outpatient clinics. Individuals aged 18 years or older, cognitive ability and preserved verbal communication were included. Patients with reports of pain or discomfort at the time of the interview and patients with a diagnosis of delirium or dementia recorded in the medical records were excluded.

### Data collection procedures

Forms elaborated by the researchers were used to collect sociodemographic and clinical information. Patients were assessed using the Karnofsky Performance Status Scale, Edmonton Symptom Assessment Scale (ESAS), Functional Assessment of Chronic Illness Therapy-Spiritual Well-Being (FACIT-sp-12) and Hospital Anxiety and Depression Scale (HADS), applied by trained interviewers.

The Karnofsky Performance Status Scale aims to assess patients’ functional capacity. The scores range from 0 to 100, with 100 functional capacity preserved and 0 patient in the death process^(14)^. The ESAS assesses the intensity of clinical symptoms, pain, fatigue, nausea, depression, anxiety, drowsiness, appetite, well-being, dyspnea, and sleep. Each score is assessed from 0 to 10, with the possibility of the overall assessment of symptoms. The total score ranges from 0 to 100 points and indicates symptom overload^(15)^.

The FACIT-sp 12 has 12 items that assess patients’ spiritual well-being, consisting of two subscales: “meaning/peace” and “faith”. There are no cut-off points established. The higher the score, the better the perception of spiritual well-being^(16)^.

The HADS consists of 14 items, subdivided into two scales for anxiety and depression, with 7 items each. Scores greater than or equal to 8 on the anxiety subscale indicate anxious symptoms, and scores greater than or equal to 9 on the depression subscale indicate the presence of depressive symptoms. The total score on each subscale is 21 points^(17)^.

### Analysis of results, and statistics

Data were entered into an Excel database and analyzed in SPSS using descriptive statistics and Spearman’s correlation analysis. Qualitative variables are presented as raw numbers and percentages and quantitative variables are presented as mean, standard deviation and median. P-values lower than 0.05 were considered significant. The parameters to assess the magnitude of the correlation were: 0 ≤ | r | <0.3 = weak correlation; 0.3≤ | r | <0.7 = moderate correlation; 0.7≤ | r | <1 = strong correlation.

## RESULTS

The study sample consisted of 135 patients under palliative care. Sociodemographic characteristic analysis showed a predominance of males (54.8%), with a mean age of 65 years, low education (mean of 6 years of study) and mean monthly income of 1.5 minimum wages. Regarding diagnosis, 68.2% of patients had neoplasms, followed by diseases of the respiratory system (11.8%), cardiovascular diseases (6.6%) and neurological diseases (6.6%).

As for performance, measured by the Karnofsky Performance Status Scale, the mean was 66.4 (16.6), i.e., patients needed occasional assistance, but were still able to carry out most of their activities. Table 1 shows the details of sociodemographic and clinical characteristics.

**Table 1:** Sociodemographic and clinical characteristics of patients, São Paulo, São Paulo, Brazil, 2018

Qualitative variables (N=135)	n (%)	
Sex		
Male	74 (54.8)	
Female	61 (45.2)	
Religion		
Catholic	76 (56.3)	
Evangelical	43 (31.9)	
Other	6 (4.4)	
Without religion	3 (7.4)	
Marital status		
With a partner	71 (52.6)	
Without a partner	64 (47.4)	
Work		
Retired	92 (68.2)	
Sick pay	18 (14.1)	
Unemployed	13 (9.6)	
Pensioner	11 (8.2)	
Diagnosis		
Neoplasms	92 (68.2)	
Diseases of the respiratory system	16 (11.8)	
Diseases of the cardiovascular system	9 (6.7)	
Neurological diseases	6 (4.4)	
Chronic kidney disease	3 (2.2)	
Others^*^	9 (6.8)	
**Quantitative variables (N=135)**	Mean (SD^**^)	**Median**
Performance (Karnofsky)	66.4 (16.6)	60
Age (years)	65.0 (16.9)	66
Education (years)	5.96 (4.7)	5
Income (minimum wage)	1.53 (1.2)	1
Diagnosis time (months)	75.4 (82.7)	39.5

* *Skin (1.5%), hematological (1.5%), liver (1.5%), rheumatic (0.7%) and congenital malformation (1.5%) diseases;*

** *SD - standard deviation.*

In the socio-demographic characterization questionnaire, patients were asked about their religiosity and 92.6% claimed to have a religious belief. Analysis of symptoms using the ESAS showed changes in well-being (65.2%), anxiety (63.7%), sadness and fatigue (both with 63%) as the most prevalent symptoms, followed by pain (52.5%).

Symptom intensity was also assessed. The most intense symptom was anxiety (mean 5.1; DS 4.3), followed by tiredness or fatigue (mean 4.9; SD 4.2) and sadness (mean 4.7; SD 4.1). Appetite change (mean 3.1; SD 3.4), sleepiness (mean 2.9; SD 3.4) and nausea (mean 2.0; SD 3.5) were the mildest symptoms among the assessed patients.

The HADS analysis indicated anxiety symptoms in 47.4% of patients and depressive symptoms in 41.5% (Table 2).

**Table 2:** Prevalence of anxiety and depression (N=135), São Paulo, São Paulo, Brazil, 2018

Anxiety scale		Depression scale	
HADS^*^		HADS^*^	
Yes (score ≥8)	n = 64 (47.4%)	Yes (score ≥9)	n = 56 (41.5%)
No (score <8)	n = 71 (52.6%)	No (score <9)	n = 79 (58.5%)
Mean (SD^**^)	7.9 (4.9)	Mean (SD^**^)	7.9 (4.6)

* *HADS - Hospital Anxiety and Depression Scale;*

** *SD - standard deviation.*

The FACIT-12 scale was used to assess spiritual well-being. The FACIT-12 total score ranges from 0 to 48 and in this study the mean was 35.4 (SD 8.8), with a median of 37. In the meaning/peace domain, a mean of 22.3 (SD 6, 1) and median 24. In the faith domain, the mean was 13 (SD 3.9) and median 14. Spiritual well-being analysis indicated moderate to high scores for the total scale and perception of meaning/peace and faith (Table 3).

**Table 3:** Distribution of spiritual well-being scores for total scale and domains (N=135), São Paulo, São Paulo, Brazil, 2018

	Mean	(SD^*^)	1^st^Q^**^	Median	3rdQ#	Min	Max
Facit§ Meaning/peace	22.4	(6.1)	19	24	28	0	32
Facit§ Faith	13.1	(3.9)	12	14	16	0	16
Facit§ Total	35.5	(8.8)	31	37	42	0	48

* *SD - standard deviation;*

** *1^st^Q - first quartile; #3^rd^Q - third quartile; Min - minimum; Maxmaximum; §Facit - Spirituality Scale.*

### Analysis of the correlation between symptoms and performance

There was a weak negative correlation between symptom overload and performance (r = -0.317; p<0.001), indicating that the lower the performance, the more symptoms the patient presents. The analysis of the correlation between performance and anxiety symptoms (r = -0.220; p=0.011) and performance and depression (r = -0.313; p<0.001) also showed a weak negative correlation, indicating that the lower the functional capacity, the more symptoms of depression and anxiety.

### Correlation analysis between symptoms and spiritual well-being

Sadness, dyspnea, drowsiness and anxiety showed a weak negative correlation with spiritual well-being. Depression, on the other hand, showed a moderate negative correlation with spiritual well-being, indicating that the greater the intensity of these symptoms, the worse spiritual well-being perception (Table 4).

**Table 4:** Correlation between spiritual well-being and symptoms in patients under palliative care (N=135), São Paulo, São Paulo, Brazil, 2018

	Facit# total (r^*^)	*p* value
ESAS§		
Anxiety	- 0.195	0.024
Sadness	- 0.309	<0.001^**^
Well-being	- 0.162	0.061
Pain	- 0.081	0.347
Tiredness	- 0.181	0.036
Nausea	- 0.079	0.362
Sleepiness	- 0.228	0.008^**^
Appetite	- 0.049	0.573
Dyspnea	- 0.289	0.001^**^
Sleep	- 0.109	0.206
Symptom overload (Total ESAS)	- 0.338	<0.001^**^
HADS^***^		
Anxiety	- 0.236	0.006^**^
Depression	- 0.469	<0.001^**^

*§ESAS - Edmonton Symptom Scale; HADS - Hospital Anxiety and Depression Scale; #Facit - Spirituality Scale;*

* r=Spearman’s correlation coefficient;

** *Statistical significance p<0.05.*

## DISCUSSION

Regarding sociodemographic variables, most participants were male, with cancer, mean age of 65 years, low education and low income. An Italian study assessing patients under palliative care also found a predominance of male patients, with a slightly higher mean age (72 years)^(18)^. As for low education and income, these results are similar to other studies with cancer patients under palliative care developed in Brazil, in which low education and income are observed^(19-20)^. It is worth mentioning that low educational level and low income are among the factors that increase the time to start treatment for colon and rectum cancer, which can impact survival^(21)^.

A study that analyzed the experience of elderly people with cancer showed that spirituality and religiosity were coping strategies used to experience suffering and uncertainties related to the illness process^(22)^. Studies conducted in Brazil show that 95% of people declared to have religion and 83% consider religion very important^(23-24)^.

The most prevalent and intense symptoms were changes in well-being, anxiety, sadness and fatigue, as in other studies^(22-25)^. In research carried out in Portugal with patients with no prospect of a cure, the most intense symptoms were anxiety, depression and fatigue^(25)^, in line with the present study.

The expression of symptoms is quite variable and depends on individual perception, among other factors. Although symptoms are addressed individually, patients often present multiple symptoms simultaneously^(26-27)^. In this regard, other studies showed emotional symptoms such as the most prevalent and intense in this population^(28-29)^.

The prevalence of anxiety and depression symptoms was high. A recent Brazilian study assessed these symptoms in cancer patients and identified 31.3% of anxiety symptoms and 26.2% of depression^(30)^. In another national study, the prevalence of anxiety symptoms was 25% and of depressive symptoms was 40%^(31)^. This study showed that patients under palliative care had higher levels of anxiety and depression than cancer patients and the general population^(30,32)^. A multicenter study conducted in a palliative care network in Germany found that women have more anxiety and fatigue than men^(33)^.

The relationship between functional capacity and physical symptoms was analyzed in the present study and a weak negative correlation was found, indicating that decreased functional capacity was followed by a greater burden of symptoms. Similar findings were observed in a study that analyzed the relationship between depression, performance and symptoms in patients with advanced cancer^(34)^. The authors concluded that the higher burden of symptoms was associated with worse performance^(34)^.

As for emotional symptoms, a discrete negative correlation was observed between functional capacity, anxiety and depression. A divergent result was found in a study with patients with breast cancer, in which no association was found between depressive symptoms and performance^(35)^.

Spiritual well-being analysis indicated moderate to high scores for the FACIT-12 scale, with a total average of 35.4 (SD 8.8), suggesting a good perception of spiritual well-being among participants. Some authors indicate religiosity as a protective factor against the development of depression, anxiety and substance abuse, often associated with better quality of life indexes^(23,36)^. The search for spirituality can contribute to the adoption of healthier lifestyles, providing social support, greater self-acceptance and resilience, factors that can contribute to psychological distress relief^(23,36)^.

In the present study, the correlation between spiritual well-being and sadness was negative, weak and significant. A study conducted in Brazil with patients with psychiatric disorders found a weak and significant negative correlation between depression and spiritual well-being^(23)^.

Spirituality has an important impact on the outcomes of patients under palliative care and their families, with positive repercussions on physical and emotional stress, reducing suicide and depression risks^(37)^. On the other hand, when the personal relationship with spirituality is negative, the effects are opposite. Research that assessed the relationship between spiritual coping and depressive symptoms, in relatives of children with cancer, concluded that coping or negative coping showed a strong relationship with depressive symptoms^(38)^. These findings indicate the relevance of the spiritual experience and the importance of health professionals addressing spirituality with patients and caregivers.

Another study sought to assess spiritual suffering and the process of re-signification through the application of relaxation, mental images and spirituality (RIME) techniques. The authors observed positive results from the technique on the quality of life of patients who found significant relief for distress^(39)^.

The data show the need to explore the field of spirituality of patients under palliative care and create intervention strategies aimed at identifying and alleviating spiritual distress. Some studies show that being present, listening carefully, facilitating encounters and demonstrating commitment to patients are some strategies of spiritual nursing care^(40)^.

There are also recent studies demonstrating connections between clusters of neuropsychological symptoms, biobehavioral manifestations and the immune, emotional and neuroendocrine systems, opening up new possibilities for developing health interventions^(41)^.

Nursing interventions for managing symptom clusters based on theories have great potential to improve the quality of care for patients under palliative care and nursing is at the forefront of these approaches^(42-43)^. In this perspective, it is expected to offer greater comprehensiveness in physical, mental and spiritual care and comfort, positively influencing the course of the disease and meeting the principles of palliative care.

### Study limitations

Since it is a descriptive correlational study, it was possible to identify relationships between the variables, but it is not possible to establish cause-effect relationships between the explored variables, in this type of study.

### Contributions to nursing

This study presents knowledge that will allow nurses to identify the most prevalent symptoms in patients under palliative care and their relationship with spiritual well-being and performance. It can also contribute to the generation of a culture that includes the spiritual dimension in the care of patients and family members in health institutions. The results reveal the need for continuing education in this area, in order to optimize care, distribute resources according to patients’ needs and promote the spiritual well-being of patients under palliative care.

## CONCLUSIONS

The most prevalent and intense symptoms were changes in well-being, anxiety, sadness and fatigue. Knowing the prevalence of symptoms and their intensity is important for nurses, because it allows better planning of care, which may reflect better results. Sadness, dyspnea, drowsiness, anxiety and depression presented a negative correlation with spiritual well-being, suggesting an association between the presence of these symptoms and impairments to spiritual well-being. Functioning also showed a negative correlation with symptom burden, indicating that effective symptom management can have repercussions on performance.

Spirituality is a significant aspect in coping with life-threatening diseases and is associated with the symptoms of patients under palliative care. Nurses should seek to control symptoms, improve spiritual well-being and performance. Further studies should investigate interventions capable of reducing symptoms and minimizing patients’ spiritual distress, helping them to achieve better performance and quality of life.

## Data Availability

https://doi.org/10.48331/scielodata.XTIU9S
